# Pulmonary Limited MPO-ANCA Microscopic Polyangiitis and Idiopathic Lung Fibrosis in a Patient with a Diagnosis of IgA Nephropathy

**DOI:** 10.1155/2015/378170

**Published:** 2015-07-22

**Authors:** Alwin Tilanus, Patricia Van der Niepen, Caroline Geers, Karl Martin Wissing

**Affiliations:** ^1^Departamento de Medicina Interna/Infectologia, Hospital General de Medellin Luz Castro de Gutiérrez, Carrera 48 # 32- 102, Medellin, Colombia; ^2^Departement Interne Geneeskunde/Nefrologie, Universitair Ziekenhuis Brussel, Laarbeeklaan 101, 1090 Brussels, Belgium; ^3^Departement Anatomo-Pathologie, Universitair Ziekenhuis Brussel, Laarbeeklaan 101, 1090 Brussels, Belgium

## Abstract

We present a case of a male patient with chronic renal insufficiency, due to crescentic glomerulonephritis with IgA deposits, who successively developed (idiopathic) thrombocytopenic purpura (ITP) and MPO-ANCA microscopic polyangiitis (MPA) with pulmonary fibrosis. The patient presented with cough, weight loss, and dyspnea on exertion. CT imaging and pulmonary function tests were compatible with interstitial pneumonitis with pulmonary fibrosis. Laboratory results showed high MPO-ANCA titers; the urinary sediment was bland. The patient was treated successfully with cyclophosphamide and methyl-prednisolone. This unique case illustrates the diagnostic and therapeutic challenges of an unusual presentation of microscopic polyangiitis presenting first as isolated kidney disease with recurrence in the form of pneumonitis without renal involvement, in association with renal IgA deposits and ITP as coexisting autoimmune conditions.

## 1. Introduction

The association between pulmonary fibrosis (PF) and Myeloperoxidase Anti-Neutrophil Cytoplasmic Antibodies (MPO-ANCAs) positivity has been reported in several small retrospective case-series, but the pathologic mechanism remains unclear [1]. In published case series [2–8] most patients were men with an average age of about 70 years, presenting typically with dry cough and dyspnea.

In a large proportion of cases, the diagnosis of MPO-ANCA vasculitis was preceded months to years by the diagnosis of PF or signs of interstitial pneumonia. During follow-up prognosis is unfavorable due to respiratory failure [2–8].

MPO-ANCAs have been reported in 7% to 33% of patients with interstitial pneumonia and idiopathic pulmonary fibrosis. Along the same line pulmonary fibrosis has been detected in one-third of a cohort of patients with p-ANCA-MPO [5, 7, 9].

Studies of patients with ANCA-positive and ANCA-negative PF demonstrated no significant differences between these clinical entities regarding symptoms, pulmonary function tests, and CT scanning [1, 5]. In most patients, more than one organ is affected, most frequently the kidney.

Here we report a unique case of MPO-ANCA microscopic polyangiitis affecting primarily the lung in a patient with a history of MPO-ANCA microscopic polyangiitis with renal-limited vasculitis and coexisting IgA deposits as well as idiopathic thrombocytopenic purpura (ITP).

## 2. Case Report

A 71-year-old man was admitted in June 2012 because of progressive dyspnea on exertion, dry cough, progressive weight loss of approximately 20 kg in 3 months, and edema of the lower extremities.

He had a complex medical history of autoimmune diseases including chronic kidney disease from a biopsy-proven crescentic glomerulonephritis with MPO-ANCA and coexistent glomerular IgA deposits in 2007 and idiopathic thrombocytopenic purpura (ITP) in 2011. The patient had never smoked.

In December 2007 he was hospitalized because of acute renal failure with serum creatinine >1060 *μ*mol/liter, oliguria, microscopic hematuria (250 RBC/*μ*L), and proteinuria (1.3 g/day). Furthermore, he had an inflammatory syndrome (CRP 194 mg/liter) and very high ANCA (>1/5120) and anti-MPO (2337 IU/mL) titers.

**Figure 1:**
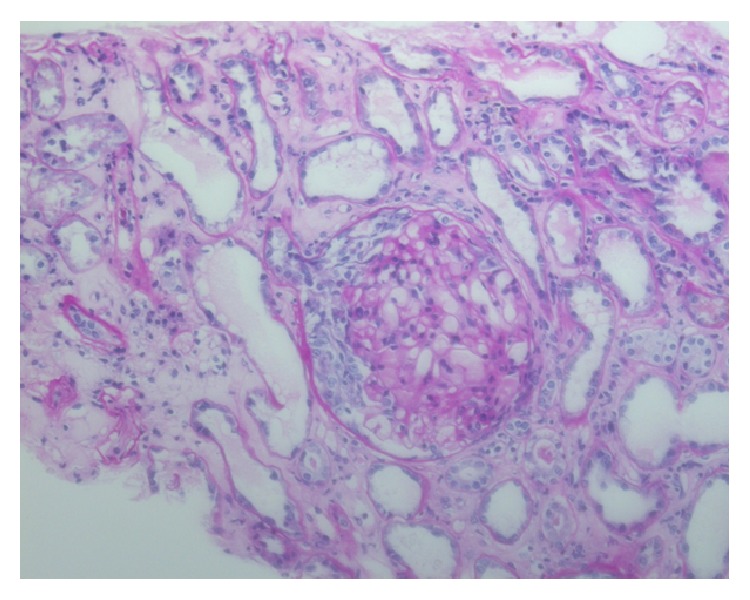
Periodic acid-Schiff stained histologic preparation with an overview of the renal parenchyma. The glomerulus shows a cellular crescent. The glomerular tuft shows mesangial hypercellularity. Tubular cells are damaged with regenerative features (PAS, ×200).

**Figure 2:**
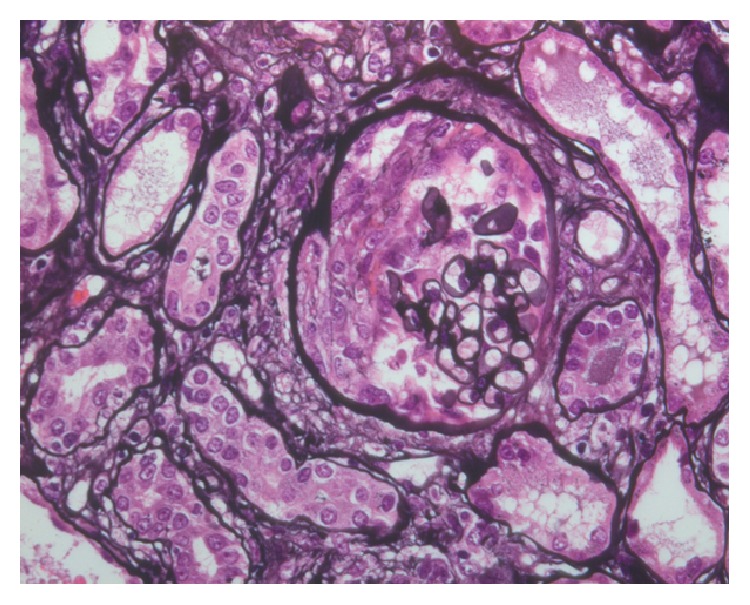
The methenamine silver stain shows fibrinoid necrosis with early crescent formation in the urinary space (Jones methenamine silver, ×400).

Renal biopsy (2007) showed necrotizing crescentic glomerulonephritis (Figures 1 and 2) with significant IgA deposits (not shown in figure).

The patient received a diagnosis of severe IgA nephropathy with crescentic glomerulonephritis and was initially treated with high dose corticosteroids and hemodialysis. His kidney function progressively recovered and he was able to stop dialysis therapy after 3 months. Between June 2008 and 2011 serum creatinine values stabilized at values between 206 and 252 *μ*mol/liter with maintenance methyl-prednisolone of approximately 8 mg every other day. Microscopic hematuria and proteinuria resolved and anti-MPO antibodies decreased from 2337 IU/mL to 208 IU/mL by April 2009.

In October 2011 the patient was hospitalized for epistaxis and severe thrombocytopenia 7 *∗* 10^3^/mm^3^ in the absence of anemia or leucopenia. The patient had no clinical signs of recurring renal disease or vasculitis and anti-MPO ANCA had further decreased to 45 IU/mL. Bone marrow examination was performed with diagnosis of idiopathic thrombocytopenia. Platelets rapidly normalized after short course of intravenous methyl-prednisolone followed by increased doses of maintenance steroids.

Interstitial pneumonia with beginning pulmonary fibrosis was diagnosed in November 2011 during evaluation for progressive dyspnea. A Broncho Alveolar Lavage (BAL) (performed under corticoid treatment) showed no abnormalities.

During the following six months his clinical condition progressively worsened in spite of treatment with high dose steroids and the patient was hospitalized several times for corticosteroid-induced diabetes, herpes zoster infection, and cellulitis of the right leg.

When admitted in June 2012 he appeared severely ill, wheelchair bound, with symmetric muscle wasting and loss of strength and resting dyspnea. He was profoundly anorectic with a loss of 27 kg since October 2011 and of 16 kg during the two month before admission. The examination of the chest revealed normal heart tones and bibasal fine lung crackles with oxygen saturation at 93%. Examination of the abdomen was unremarkable. Venous insufficiency and slight pitting edema were present at both lower limbs.

Laboratory examination showed a normocytic anemia Hb 7.0 g/dL, normal leukocyte count, an elevated C-reactive protein (CRP 246 mg/liter), and erythrocyte sedimentation rate (ESR 99 mm/h). Serum creatinine was stable at 203 *μ*mol/liter; the urinary sediment showed no red or white blood cells and no casts; no proteinuria was detected. The patient had normal levels of eosinophils.

As compared to December 2011 and in spite of high dose steroids, p-ANCA titers had increased from 1/80 to 1/640 U/mL and MPO antibodies were above the upper limit of the titration curve of the test.

During the same period, spirometry showed a remarkable worsening of the restrictive pattern with forced expiratory volume in 1 second (FEV1) decreasing from 1.99 L to 0.71 L and diminishing carbon monoxide (CO) diffusion capacity of the lung.

**Figure 3:**
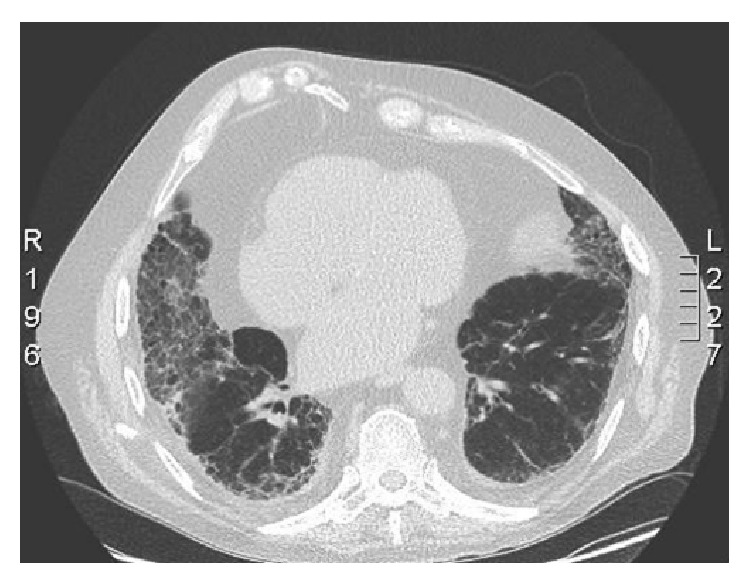
CT scan (prior to treatment) showing diffuse pulmonary fibrosis (honeycombing) and bilateral pleural fluid collection.

CT scan of the thorax was classified as usual interstitial pneumonia (UIP) with reticular infiltrates and honeycombing predominantly in the subpleural areas of the lower lobes, without possibility to differentiate between idiopathic pulmonary fibrosis and fibrosis secondary to vasculitis on radiological grounds (Figure 3). Giant vessel vasculitis was excluded by PET-CT scan (though performed under corticosteroid treatment). A biopsy of the nasal mucosa showed nonspecific inflammation without signs of vasculitis or granulomatous lesions. There were no signs of active renal disease with a bland urinary sediment and absence of proteinuria as well as a stable but decreased renal function (CKD stage 3B).

Because of inflammation in the blood and a sputum culture positive for Moraxella/Proteus, the patient was treated with amoxicillin/clavulanic acid. This treatment was complicated by the development of an extensive erythematous skin rash spreading over the entire body. Skin biopsies showed diffuse infiltrates with eosinophils and neutrophils and were protocoled as drug-induced rash. Antibiotic treatment was changed to moxifloxacine and 32 mg/day methyl-prednisolone with improvement of the rash. At that time the patient was considered too ill to undergo open lung biopsy for a formal histological diagnosis of fibrosis and/or vasculitis. In the context of increasing ANCA, an inflammatory syndrome as well as anorexia and severe weight loss microscopic polyangiitis with rapidly progressive pulmonary fibrosis was considered as sufficiently likely to justify the initiation of combination therapy with intravenous cyclophosphamide and high dose oral corticosteroids according to the CYCLOPS protocol [10].

Within days after the first cycle of cyclophosphamide we observed remarkable clinical improvement with decrease in dyspnea, increased appetite, and improved gait.

After 5 cycles of IV cyclophosphamide, pANCA/MPO titers decreased from 1/640 to 1/320 and 134 to 44 U/mL, respectively, and inflammatory parameters normalized. Furthermore, FEV1 significantly improved from 0.71 to 1.63 L, Table 1. The CT scan showed no changes. The patient was last seen at the outpatient clinic in December 2012 with a serum creatinine of 190 *μ*mol/L, hemoglobin levels of 11.4 g/dL, and a CRP of 8.6 mg/L. Weight increased from 81 to 91 kg during the 6 months after starting cyclophosphamide and the patient was again able to walk without assistance. He received a maintenance immunosuppression with azathioprine and Medrol 4 mg/day and no longer returned for follow-up at our unit.

## 3. Discussion

The present case is characterized by both the unusual association of several autoimmune conditions and a sequential and atypical presentation of microscopic polyangiitis, with selective damage of first the kidneys and subsequently the lungs without recurring of kidney disease. The initial presentation of our patient with rapidly progressive renal failure, high titers of MPO-ANCA, and crescentic glomerulonephritis with fibrinoid necrosis was clearly suggestive of renal-limited microscopic polyangiitis. The patient received a diagnosis of aggressive IgA nephropathy at this moment because of the presence of mesangial IgA deposits. Similar to our patient, several reports have described patients with (crescentic) IgA nephropathy in the presence of positive ANCAs [11–14]. It is unclear whether IgA nephropathy precedes ANCA positivity or if mesangial inflammation due to IgA deposits creates an inflammatory microenvironment that triggers vasculitis. Similar to our patient the clinical presentation is in general characterized by rapid loss of renal function and requires aggressive immunosuppressive therapy combining cyclophosphamide and steroids. Although our patient ultimately entered remission and was able to stop dialysis therapy, in retrospect we have to conclude that he should have been treated for MPA at the first presentation of his disease.

Thrombotic and thrombocytopenic purpura (TTP) has been described in rare patients with ANCA-associated vasculitis [15–17]; there has been, to the best of our knowledge, no previous report on the association between idiopathic thrombocytopenic purpura and ANCA-associated vasculitis. The relation of ITP with vasculitis in our patient is not clear because it occurred at a moment when MPO-ANCA titers were low but ITP was also rapidly followed by recurrence of vasculitis in the form of pulmonary fibrosis. Severe thrombocytopenia rapidly responded to high dose steroids and did not recur during the rest of follow-up.

Our patient developed progressive interstitial pneumonitis and pulmonary fibrosis that was first diagnosed approximately one month after the development of ITP and occurred in spite of the administration of high dose steroids for ITP and initially without increase in MPO-ANCA and other signs of MPA. The link with ANCA vasculitis was made only 6 months later when the patient had developed severe systemic disease in the form of anorexia, wasting, anemia, and an inflammatory syndrome in the context of high MPO-ANCA titers. Surprisingly, although MPA had initially presented as renal limited disease, the recurrence occurred without any signs of renal involvement. The presentation of our patient is compatible with the largest series published up to now which reported a predominance of older male patients with MPO-ANCA among those developing PF and a majority presenting with a typical UIP pattern on CT scans [8]. Homma et al. examined 31 patients diagnosed as having PF with positive MPO-ANCA. In 11 autopsied patients the histopathological features of the diseased lung tissues were compatible with the usual interstitial pneumonia pattern. Vasculitis in bronchial arteries and/or pulmonary arterioles was confirmed in only five patients [6]. Hervier et al. reported low diagnostic yield of 3 transbronchial biopsies which showed only fibrosis and no vasculitis [4]. On the contrary a large series of patients with open lung biopsy (25) or autopsy (2) provided a correct diagnosis of vasculitis in 21 of 27 ANCA positive patients (78%), capillaritis being the most common lesion [18]. In the present patient we decided against transbronchial biopsy because of the low diagnostic yield and against open lung biopsy because the patient was considered too ill to undergo surgery. The treating physicians had also decided that systemic inflammation with anorexia and wasting in the context of high titers of MPO-ANCA were a justification of standard treatment of MPA irrespective of the results of an eventual open lung biopsy.

The pathologic mechanism of pulmonary fibrosis and positive MPO-ANCA remains unclear [1]. There is evidence that infection and certain drugs can stimulate ANCA-MPO/PR3 antibody production [19]. Infection can prime and activate neutrophils by circulating inflammatory cytokines. Subsequent translocation of ANCA antigens (e.g., MPO/PR3) to the cell surface of neutrophils and expression of adhesion molecules by endothelial cells result in cell adhesion, release of reactive oxygen species, vasculitis, and endothelial apoptosis [20]. Chan et al. described a case of MPO-ANCA microscopic vasculitis following a suppurative wound infection in a cancer patient [21]. Infectious events in our patient (cellulitis right leg, pneumonia) might have triggered MPO-ANCA titers increase. Takato et al. described a case of an MPO-ANCA positive PF patient in which a Mycoplasma infection triggered the elevation of MPO-ANCA titers and the development of a crescentic glomerulonephritis [22].

When examining the published case series [2–8], prognosis was typically worse when PF was associated with MPO-ANCA positivity especially when eosinophilia was present [4]. The MPO-ANCA antibodies could lead to pulmonary capillaritis which might result in (subclinical) alveolar hemorrhage and finally fibrosis [23–26]. Nozu et al. suggested that there is some evidence that corticosteroid therapy is more effective in ANCA positive patients and that survival tends to be better when the MPO titers were low (<50 IU/L) [5]. Nevertheless, and similar to the initial clinical presentation of our patient who developed fibrosis before increasing MPO titers, the development of PF has been reported to precede increase in MPO-ANCA positivity. In about half of the patients the diagnosis of PF precedes the diagnosis of vasculitis [8]. Therefore the opposite theory that PF could result in MPO-ANCA production and be a trigger for the development or recurrence of MPA cannot be discarded [4, 7]. The reported lack of clear correlation between MPO-ANCA titers and severity of PF also question the unique role of ANCA in the disease process [6].

Although in our case the lungs were the only vital organ affected, other organs might have been subsequently damaged during disease progression as previously reported by Hiromura et al. In this report, four patients with MPO-ANCA positive idiopathic pulmonary fibrosis subsequently developed rapidly progressive glomerulonephritis [27].

Our patient was treated according to the vasculitis guidelines (CYCLOPS protocol [10]) with rapid clinical improvement. Renal function improved and MPO-ANCA titers decreased significantly. A control spirometry after 5 cycles of cyclophosphamide showed a remarkable improvement in total lung volume and FEV1. However, a control pulmonary CT scan was unchanged, indicating that most lung tissue damage was irreversible.

Since high (e.g., >50 IU/mL) MPO-ANCA titers in a patient with PF carry a worse prognosis and risk of evolution to MPA, every patient with (idiopathic) PF should be screened for MPO-ANCA positivity. When positive, we propose to look for underlying infections, treat them as necessary, and start treatment with cyclophosphamide/corticosteroids, even without prior lung biopsy.

## Figures and Tables

**Table 1:** Change of p-ANCA, MPO, spirometry, and CT scanning characteristics in the period from December 2011 to October 2012.

	Period		
	December 2011	June 2012	October 2012 (after 5 cycles of cyclophosphamide)
p-ANCA	1/180	1/640	1/320
MPO (IU/L)	45	>134^1^	44
*Spirometry *			
FVC (forced vital capacity) (liters)	2.51 (58% of PV^2^)	0.84 (20% of PV)	2.23 (52% of PV)
Forced expiratory volume 1 second (FEV1) (liters)	1.99 (61% of PV)	0.71 (22% of PV)	1.63 (50% of PV)
FEC/FVC %	79 (74% of PV)	84% (74% of PV)	73% (74% of PV)
VC (vital capacity) (liters)	2.51 (56% of PV)	0.98 (22% of PV)	2.23 (50% of PV)
TLC (total lung capacity) (liters)	3.84 (51% of PV)	Not measured	3.96 (53% of PV)
RV (residual volume) (liters)	1.33 (49% of PV)	Not measured	1.73 (63% of PV)
DLCO (diffusing capacity of the lung for carbon monoxide) (mL/mmHg/min)	9.2 (32% of PV)	Not measured	7.6 (27% of PV)
CT scanning	Interstitial pneumonia/idiopathic lung fibrosis	Interstitial pneumonia/idiopathic lung fibrosis	Unchanged (permanent damage)

^1^Protocolled as >134 IU (above upper limit of test range).

^2^PV: Predicted Value.
